# Creatine Supplementation Combined with Exercise in the Prevention of Type 2 Diabetes: Effects on Insulin Resistance and Sarcopenia

**DOI:** 10.3390/nu17172860

**Published:** 2025-09-03

**Authors:** Ewelina Młynarska, Klaudia Leszto, Kinga Katańska, Aleksandra Prusak, Anna Wieczorek, Paulina Jakubowska, Jacek Rysz, Beata Franczyk

**Affiliations:** 1 Department of Nephrocardiology, Medical University of Lodz, ul. Zeromskiego 113, 90-549 Lodz, Poland; 2 Department of Nephrology, Hypertension and Family Medicine, Medical University of Lodz, ul. Zeromskiego 113, 90-549 Lodz, Poland

**Keywords:** exercise, creatine, type 2 diabetes, insulin resistant, skeletal muscle mass, sarcopenia

## Abstract

Type 2 diabetes (T2D) is a chronic metabolic disorder marked by insulin resistance and impaired glucose metabolism, with skeletal muscle being a major site of systemic glucose disposal. This review examines the bidirectional relationship between T2D and sarcopenia, and synthesizes current evidence on how skeletal muscle deterioration and insulin resistance interact to disrupt glucose homeostasis. We summarize molecular mechanisms by which physical exercise enhances glucose uptake via insulin-dependent and insulin-independent pathways, and review the ergogenic and metabolic effects of creatine monohydrate (CrM). We also evaluate studies combining CrM supplementation with resistance or aerobic training and their effects on glycaemic control, muscle mass and function. Overall, combined exercise and creatine supplementation show potential to improve glucose regulation and attenuate muscle loss in older adults and people with T2D. Available data indicate that CrM is well tolerated in healthy and clinical populations when used at recommended doses, with no consistent evidence of adverse renal or hepatic effects. Further large randomized trials are needed to define optimal dosing, training modalities and long-term benefits for metabolic outcomes.

## 1. Introduction

Persistent hyperglycemia is a hallmark of type 2 diabetes (T2D), a chronic metabolic disease. It may result from decreased insulin secretion, reduced sensitivity to insulin in peripheral tissues (insulin resistance), or a combination of both [1,2]. In the early stages, the pancreas compensates for insulin resistance by increasing insulin production. However, over time, this compensatory mechanism fails, insulin levels decline, and T2D develops [3].

Although T2D has traditionally affected adults over the age of 45, its prevalence is increasing among children, adolescents, and young adults, driven by rising rates of obesity, sedentary lifestyles, and consumption of energy-dense foods [4].

According to the International Diabetes Federation (IDF) Diabetes Atlas 2025, an estimated 11.1% of the global adult population aged 20–79 years is currently living with diabetes. The most significant increases have been observed in low- and middle-income countries (LMICs), where access to diagnosis and treatment remains limited [5]. Globally, nearly 500 million individuals are currently living with T2D, and its prevalence doubled between 1990 and 2022 [6,7]. Projections estimate that by 2050, the total number of people living with diabetes will reach 853 million [5].

Beyond its direct connection to insulin resistance, T2D is increasingly recognized as closely linked with musculoskeletal decline, particularly sarcopenia—the progressive loss of skeletal muscle mass and strength, which frequently coexists with T2D [8,9]. This coexistence carries important clinical implications, as sarcopenia reduces muscle-mediated glucose uptake, thereby worsening insulin resistance [10]. Conversely, insulin resistance impairs anabolic signaling and promotes muscle catabolism, accelerating muscle loss [11,12]. Such a bidirectional relationship worsens metabolic health and increases the risk of T2D progression [13].

Epidemiological studies consistently demonstrate a high prevalence of sarcopenia among individuals with T2D. In a hospital-based study of 334 patients with T2D, 30.2% were diagnosed with sarcopenia [14]. Similarly, a cross-sectional analysis of 6381 individuals over the age of 50 reported sarcopenia in 28% of participants with T2D compared with 16% in non-diabetic individuals [15]. These findings suggest a non-random association, underpinned by shared pathophysiological mechanisms, such as insulin resistance, chronic inflammation, and mitochondrial dysfunction [16].

This overlap opens a potential therapeutic target. Interventions aimed at preserving or increasing muscle mass, such as resistance training, adequate protein intake, and anabolic agents, have shown potential in improving insulin sensitivity and glycemic control [16,17]. For example, a meta-analysis of supervised aerobic exercise programs (>12 weeks) found that 87.5% of interventions led to reductions in glycosylated hemoglobin (HbA1c) [18]. In other studies, 10 weeks of resistance training in patients with T2D resulted in an 18% reduction in HbA1c [19].

**Figure 1:**
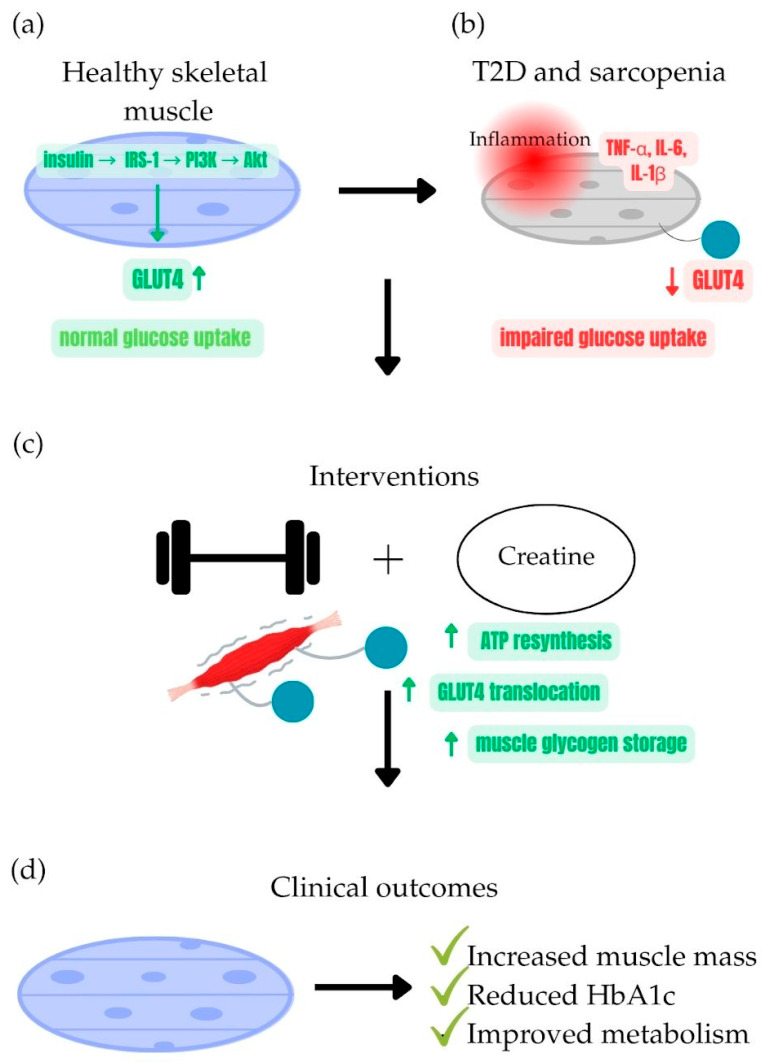
Synergistic effects of creatine supplementation and exercise on glucose metabolism and sarcopenia prevention in type 2 diabetes. (**a**) Healthy skeletal muscle: adequate muscle mass with efficient insulin-mediated GLUT4 translocation for glucose uptake. (**b**) Type 2 diabetes with sarcopenia: reduced muscle mass, chronic inflammation, impaired glucose uptake, and mitochondrial dysfunction. (**c**) Interventions: combined resistance/aerobic exercise and creatine monohydrate supplementation increase GLUT4 expression, glycogen storage, and mitochondrial activity while reducing oxidative stress. (**d**) Clinical outcomes: improved muscle strength and mass, enhanced glucose metabolism, and reduced HbA1c levels.

Integrating muscle-preserving strategies into diabetes management may therefore offer significant added value. Given the central role of muscle in glucose metabolism, creatine supplementation, a well-established tool in sports nutrition, has attracted growing interest for its potential role in improving insulin sensitivity and counteracting sarcopenia [20,21]. A schematic summary of the proposed mechanisms linking creatine and exercise to improved muscle and metabolic outcomes is presented in Figure 1.

A 2011 study showed that individuals with T2D who combined creatine supplementation with exercise experienced greater improvements in insulin sensitivity compared with those who exercised without supplementation. Creatine supplementation, as demonstrated in this study, can improve glycemic control by enhancing GLUT-4 translocation in skeletal muscle. This effect occurred without between-group differences in muscle mass or strength, suggesting a mechanism independent of hypertrophy [22]. Together, these data support a dual perspective for T2D management: (1) interventions that counteract sarcopenia can improve glycemic control, and (2) creatine may provide additional benefit even when hypertrophy is limited by enhancing glucose transport in skeletal muscle when used alongside exercise [23,24,25].

Given the association between increased skeletal muscle mass, improved glycemic control, and enhanced insulin sensitivity, creatine represents a promising adjunct in T2D management and in reducing diabetes risk, especially when combined with exercise.

Despite promising evidence, the potential of creatine supplementation in T2D management remains limited, particularly in the context of sarcopenia.

The aim of this narrative review is to analyze the interplay between insulin resistance and sarcopenia in the pathophysiology of T2D, and to evaluate the therapeutic potential of muscle-preserving interventions—particularly exercise and creatine supplementation—in managing T2D and preventing its progression.

Building on prior studies, we add and integrate several significant elements: (1) an integration of bidirectional link between sarcopenia and T2D and an appraisal of the creatine–exercise combination as a muscle preserving adjunct in metabolic care, including discussion of molecular and pathophysiological mechanisms underlying creatine’s effects, alongside evidence on its impact on sarcopenia, glucose homeostasis, and insulin resistance; (2) a synthesis of trials on creatine-exercise combinations and their effects in patients with T2D, older adults, and healthy volunteers, summarized in a single table; (3) a clinical interpretation of the safety profile, including dosing strategies; and (4) an assessment of creatine forms and co-ingestion interactions (e.g., caffeine, selected drugs) relevant to metabolic care.

## 2. The Role of Creatine in Energy Metabolism, Sarcopenia, and Its Impact on Glucose Metabolism

### 2.1. The Role of Creatine in Energy Metabolism

Creatine, chemically known as *N*-aminoiminomethyl-*N*-methylglycine, is primarily synthesized endogenously in the kidneys and liver from the amino acids arginine and glycine. Approximately 50% of the daily creatine requirement is supplied by endogenous synthesis, with the remainder obtained from diet or supplementation. About 95% of creatine in the body is stored in skeletal muscles, primarily as phosphocreatine (PCr), which, in conjunction with creatine kinase (CK), facilitates ATP regeneration—the primary energy carrier in cells. Creatine is transported into cells via specific transporters (CRTRs) and functions additionally as an antioxidant, mitigating mitochondrial oxidative stress. Between 1–2% of muscle creatine is converted into creatinine and subsequently excreted in urine; this conversion positively correlates with increased muscle mass and physical activity [26].

Besides facilitating rapid ATP regeneration during high-intensity exercise, PCr supports intracellular transport of high-energy phosphates between synthesis and utilization sites. It participates in buffering intracellular acidification by consuming hydrogen ions [27].

### 2.2. Creatine in Sarcopenia and Its Effect on Glucose Metabolism

Sarcopenia is characterized by progressive loss of muscle mass, strength, and functional capacity [28]. It primarily affects elderly individuals, with a multifactorial etiology involving factors such as physical inactivity, chronic diseases, genetic predisposition, decreased caloric intake, hormonal imbalance, and metabolic disturbances [29]. Increasing evidence suggests that sarcopenia also impacts glucose metabolism, contributing to insulin resistance and T2D [28].

Aging is accompanied by an imbalance between anabolic and catabolic muscle protein pathways, resulting in net muscle mass loss. A decrease occurs in both the size and number of muscle fibers, particularly type II fibers, owing to their transition into type I fibers, alongside infiltration by intra- and inter-muscular fat and a reduction in type II fiber satellite cells. Pathogenic crosstalk between adipose tissue and muscle is also significant. Dysfunction of adipose tissue, including adipocyte hypertrophy, overproduction of lipids and adipokines, and chronic inflammation, leads to lipid accumulation in muscles, mitochondrial dysfunction, oxidative stress, and insulin resistance. Adipose tissue and muscle communicate through cytokines, chemokines, adipokines, and myokines, creating a self-perpetuating inflammatory–metabolic loop that promotes the development of sarcopenic obesity [30].

Molecularly, mitochondrial dysfunction is characterized by excessive reactive oxygen species (ROS) production, AMP-activated protein kinase (AMPK) pathway activation, and reduced expression and activity of peroxisome proliferator-activated receptor gamma coactivator 1 alpha (PGC-1α). Consequently, chronic activation of nuclear factor kappa-B (NF-κB) and p38 mitogen-activated protein kinase (p38 MAPK) pathways occurs, leading to prolonged muscle protein degradation and weakness.

Chronic inflammation, marked by increased pro-inflammatory cytokines (e.g., tumor necrosis factor alpha (TNF-α), interleukin-1 beta (IL-1β)), signal transducer and activator of transcription 1/3 (STAT1/3) pathway activation, and excessive glucocorticoid receptor stimulation, promotes muscle fiber damage and impaired regeneration. Hormonal disturbances, such as insulin-like growth factor 1 (IGF-1) resistance, dysfunction of the growth hormone (GH)/IGF-1 axis, and impaired androgen signaling, further hinder muscle regeneration and adaptation. Moreover, heightened proteolytic activity (e.g., caspase-3-mediated), suppression of protein synthesis via mechanistic target of rapamycin complex 1 (mTORC1) signaling attenuation, satellite cell dysfunction, and cytoskeletal alterations accelerate muscle atrophy progression [31].

Regarding carbohydrate metabolism, reduced skeletal muscle mass diminishes the body’s capacity to take up and store glucose. This leads to insulin resistance and hyperglycemia [28]. Muscles produce myokines released during physical activity, which support glucose metabolism by enhancing insulin sensitivity and additionally exert anti-inflammatory effects, inhibit lipid accumulation, and counteract sarcopenia progression, thereby reducing the risk of type 2 diabetes [32].

Creatine supplementation may effectively preserve muscle mass and function in older adults, thereby playing a key role in sarcopenia prevention and mitigating its adverse health impacts [33]. Additionally, creatine may contribute to blood glucose regulation, important for preventing T2D in elderly populations. Clinical evidence remains limited, comprising a few short-term studies insufficient to support widespread practical application of creatine supplementation. In the review by Solis M. et al., it was emphasized that basic research suggests possible mechanisms, including creatine’s potential to stimulate insulin secretion in vitro and increase muscle glycogen stores, with the most effective intervention combining supplementation with physical exercise, thereby enhancing glucose transport to the sarcolemma via translocation of glucose transporter type 4 (GLUT4) [34].

## 3. The Role of Physical Exercise in Improving Glucose Metabolism

### 3.1. Molecular Mechanisms of Glucose Regulation in Skeletal Muscle

Skeletal muscle plays a critical role in the maintenance of postprandial glucose homeostasis, with over 80% of glucose being transported into skeletal muscle via insulin-dependent mechanisms [35,36,37]. Under hyperglycemic conditions, this proportion may increase to over 90% of insulin-mediated glucose uptake [38].

Following a meal, elevated insulin levels bind to insulin receptors, triggering phosphorylation of the insulin receptor substrate (IRS) and activation of the protein kinase B (Akt/PKB) pathway. As a result, GLUT4 is translocated from the cytosol to the sarcolemma, facilitating glucose entry by facilitated diffusion [35].

In addition to insulin signaling, skeletal muscle contractions promote GLUT4 translocation via distinct, insulin-independent mechanisms, including Ras-related C3 Botulinum Toxin substrate 1 (Rac1)/actin and Ca^2+^/calmodulin-dependent protein kinase (CaMK), thereby enhancing glucose uptake during and after exercise [35,39].

In patients with T2D, both GLUT4 abundance and its translocation are compromised due to insulin resistance. Overnutrition, resulting in lipid overload, reduces insulin-stimulated glucose disposal [40,41]. Moreover, lipid intermediates accumulate and act as signaling molecules influencing skeletal muscle peroxisomal metabolism, oxidative phosphorylation and insulin signaling [42]. Furthermore, they enhance the activity of protein kinase Cθ, causing inhibitory serine phosphorylation of the insulin receptor and IRS, which suppresses their function [43].

Insulin-dependent GLUT4-mediated glucose uptake is severely impaired in insulin-resistant skeletal muscle. Nevertheless, exercise-induced glucose clearance is relatively preserved in individuals with T2D, highlighting its potential importance in novel diabetes management strategies [44]. Notably, endurance exercise training promotes an increase in the amount of GLUT4 transporter within skeletal muscles, thereby improving glucose uptake and overall muscle metabolism [39].

While impairments in insulin signaling and GLUT4 translocation significantly contribute to disrupted glucose uptake in skeletal muscles in T2D, it is important to emphasize that mitochondrial dysfunction also plays a pivotal role in the pathophysiology of insulin resistance.

In the context of T2D, the nutrient overload triggers high protonmotive force and oxidative stress [45], and creates a high H2O2-emitting potential [46]. The redox imbalance resulting from an increase in ROS levels or insufficient endogenous antioxidant capacity seems to play a crucial role in the development and progression of T2D [47].

T2D has been associated with mitochondrial abnormalities, including reduced size, morphological damage, and impaired functionality [48]. Meex et al. demonstrated that combined aerobic and resistance training lasting 12 weeks led to a recovery of mitochondrial function approaching the values seen in healthy control subjects. In individuals with T2D, this mitochondrial improvement was accompanied by a partial enhancement in insulin-stimulated glucose uptake and a full normalization of both metabolic flexibility and insulin-driven substrate oxidation to values comparable to those of controls [49]. Regular physical activity modulates the oxidative balance in cells and tissues by enhancing antioxidant defense and minimizing oxidative damage induced by ROS [50].

### 3.2. Role of Exercise in Type 2 Diabetes Management

Physical activity represents a first-line strategy in the prevention and management of T2D. According to World Health Organization (WHO) Guidelines on Physical Activity and Sedentary Behaviour [51], adults living with T2D are advised to engage in either 150–300 min of moderate-intensity aerobic physical activity or 75–150 min vigorous-intensity aerobic physical activity per week, complemented by muscle-strengthening resistance training on two or more days per week. Exceeding the recommended values is associated with additional health benefits.

Aerobic exercise is defined as sustained and rhythmic physical activity that involves large muscle groups and can be performed continuously over an extended period [52,53]. Aerobic exercises include activities such as walking, cycling, or swimming. Numerous scientific studies support the efficacy of aerobic training in improving glucose regulation, specifically fasting glucose levels and HbA1c [54]. The population-based cohort study by Cuthbertson et al. assessing the correlation between the number of steps per day and the risk of developing T2D showed that accumulating more steps/day and walking with increased stepping pace contribute to a lower risk of developing T2D [55]. This finding seems consistent with the prospective cohort study on physical activity patterns and T2D incidence by Liao et al. The study examined the relationship between the duration and consistency of moderate-to-vigorous physical activity and the risk of T2D progression. The results show that a longer duration of physical activity is associated with a gradual reduction in the T2D incidence both for the regular activity group and weekend warriors. Moreover, exceeding the guideline-recommended physical activity levels results in further risk reduction [56]. Similar conclusions can be drawn from the population-based prospective cohort study conducted by Li et al., which demonstrated that both the volume and intensity of physical activity are inversely associated with T2D. While higher overall physical activity volume reduces T2D risk, engaging in higher-intensity physical activity provides additional protective benefits. Furthermore, vigorous physical activity offers the greatest benefit in lowering T2D incidence [57]. It is therefore unsurprising that aerobic training is the most frequently advocated form of exercise in physical activity guidelines worldwide [58].

While aerobic training is widely endorsed in physical activity recommendations, only a subset of standards identifies strength exercise as an important factor influencing glucose homeostasis [58]. Resistance training comprises movements performed against external resistance, such as free weights, weight machines, body weight or resistance bands [52]. The studies indicate that resistance training induced an increase in mitochondrial content [59], glucose extraction, and GLUT4 density [60] in trained muscles in individuals with T2D. The controlled trial conducted by Chien et al. showed that a 12-week strength exercise program led to improvements in HbA1c, extremity strength, and muscle mass in patients with T2D and possible sarcopenia [61]. A notable correlation has been observed between enhanced muscle strength from resistance training and glucose homeostasis. The meta-analysis of 20 studies revealed that improvements in muscular strength due to training significantly influenced HbA1c, with larger gains correlating with greater reductions [62].

### 3.3. Combined Training as an Optimal Intervention Strategy

Although both aerobic and resistance training independently improve glycemic regulation, evidence suggests that a combined approach is more effective. This potential additive effect of aerobic and strength exercise has been supported by a meta-analysis of 37 studies. Various exercise modalities, including combined exercise, supervised aerobic, supervised resistance, unsupervised aerobic, and unsupervised resistance, were compared in the analysis. The results show that combined training has the greatest potential in the reduction of HbA1c [63]. Comparable results come from controlled trials performed by Amaravadi et al. The intervention group participated in a 12-week structured training program including walking and strength exercises for both upper and lower limbs, while the control group was advised to follow standard hospital care. The study group demonstrated significant improvements in metabolic outcomes, including insulin resistance, fasting, and postprandial glucose levels and HbA1c, which support the conclusion that combined training is an effective way to maintain glucose homeostasis [64]. These results correspond with those reported by Liu et al., who demonstrated a progressive decrease in glucose values in study groups undergoing combined exercise training. The exercise group showed improved glucose stability, with values maintained below the baseline [65].

Skeletal muscle plays a central role in glucose homeostasis, and its insulin resistance is the initial metabolic impairment in the development of T2D [38]. Both aerobic and resistance training independently improve metabolic outcomes [52,53,54,55,56,57,58,59,60,61,62]. However, combined exercise programs seem to offer the greatest benefit in terms of glycemic control, insulin sensitivity, and mitochondrial function [63,64,65]. These findings support the implementation of structured, multimodal physical activity as a cornerstone in the prevention and management of T2D.

## 4. Creatine and Exercise—Synergistic Effects in the Prevention of Type 2 Diabetes

Physical activity plays a crucial role in the management of diabetes, alongside pharmacological treatment and diet. Regular exercise enhances insulin-independent glucose uptake in skeletal muscle, improves insulin sensitivity, and increases muscle glycogen stores [22,66,67,68,69,70,71,72,73].

Creatine is a widely used and common supplement among athletes and physically active individuals. It supports muscle mass, muscle strength, performance, and recovery [74,75,76,77,78,79]. Creatine increases PCr levels in muscles, which accelerates the regeneration of ATP, and this is the primary energy source during short, high-intensity efforts. This enhanced energy availability enables greater maximal strength and power output during exercise [80,81,82,83,84]. Additionally, creatine influences cellular hydration status, glycogen storage, calcium and protein metabolism, oxidative stress levels, inflammatory responses, stimulation of satellite cell activity, activation of myogenic regulatory factors, and the regulation of IGF-1 [85,86], which may contribute to the progressive enhancement of muscle tissue [87].

**Figure 2:**
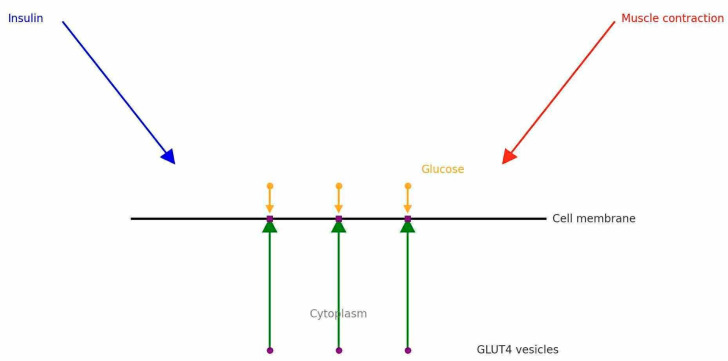
GLUT4 activation by insulin and muscle contraction.

The combination of creatine supplementation with physical exercise can act synergistically in the prevention of T2D and sarcopenia through several mechanisms compared with each intervention used separately. One of these mechanisms is the increased glucose transport into muscle cells through the translocation of GLUT4 to the sarcolemma [34]. This transporter is sequestered intracellularly in the non-stimulated state. In response to insulin or muscle contraction, it translocates to the plasma membrane, resulting in glucose uptake and normalization of blood glucose levels (Figure 2) [88,89].

### Clinical Evidence and Mechanistic Mediators

We identified randomized controlled trials combining creatine supplementation with exercise through a structured search of PubMed, using the keywords creatine, exercise, resistance training, T2D, and older adults. We included studies in adults with T2D, older adults, and experimental models relevant to these populations. Studies without a control group, without the combination of creatine and exercise, or conducted exclusively in animals/in vitro were excluded. In the following section, we discuss these studies, with their summary provided in Table 1.

Op’t Eijnde et al. conducted a double-blind, placebo-controlled trial with 22 healthy volunteers undergoing a two-week limb immobilization period. Participants were divided into two groups: one received creatine supplementation, while the other received a placebo. After immobilization, all participants underwent a 10-week rehabilitation program. The immobilization period caused a significant reduction in GLUT4 protein expression in the control group, whereas no such reduction occurred in the creatine group. Moreover, a significantly increased level of GLUT4 was observed in the creatine group during the rehabilitation phase. By the end of the 10-week recovery period, the creatine group exhibited approximately 40% higher GLUT4 expression compared with the placebo group. The researchers concluded that creatine supplementation helps prevent the reduction in muscle GLUT4 protein levels during immobilization and promotes an increase in GLUT4 content during rehabilitation training in healthy individuals [23]. Details of randomized and controlled trials combining creatine supplementation with exercise are presented in Table 1.

Similarly, Derave et al. examined the effects of creatine and creatine plus protein supplementation on GLUT4 and glycogen content of human skeletal muscle. The right legs of 33 healthy participants were immobilized for two weeks, followed by a six-week resistance training program for the right knee extensor muscles. Participants were supplemented throughout the study with either a placebo, creatine, or creatine during immobilization and creatine plus protein during retraining. The researchers concluded that creatine (especially when combined with protein) enhances GLUT4 recovery and upregulation and glycogen content, but only when paired with increased activity levels [90].

Gualano et al. evaluated the effects of creatine supplementation on glucose tolerance and insulin sensitivity in sedentary, healthy males undergoing aerobic training. In this randomized, double-blind, placebo-controlled trial, 22 volunteers received either creatine or a placebo while simultaneously participating in a moderate-intensity aerobic exercise program. The results demonstrated a significantly enhanced reduction in plasma glucose levels in response to the oral glucose tolerance test (OGTT) in the creatine group compared with the placebo group, but there were no differences in fasting insulin or homeostatic model assessment of insulin resistance (HOMA-IR).

In another trial, Oliveira et al. conducted a randomized, double-blind, placebo-controlled trial involving community-dwelling older adults. Participants were assigned to one of two groups: creatine supplementation combined with resistance training, or placebo combined with resistance training, and they completed a 12-week resistance training program. As with the previous study, improvements in fasting insulin and HOMA-IR were not observed, suggesting a potential additive effect of creatine on glycemic control, but not on insulin resistance [92].

The most compelling evidence comes from the study conducted by Gualano et al., which was a 12-week, randomized, double-blind, placebo-controlled clinical trial designed to evaluate the effects of creatine supplementation combined with exercise on glycemic control in individuals with T2D. A total of 25 adults with diabetes were randomly assigned to receive either creatine monohydrate (CrM) or a placebo while undergoing an exercise program consisting of aerobic and resistance training sessions, performed three times per week. The researchers observed that creatine supplementation, along with exercise training, significantly reduced HbA1c and glycemia in response to a meal tolerance test. No significant differences were observed for insulin and C-peptide concentrations. This study suggests that the beneficial effect on glycemic control appears to be associated with enhanced GLUT4 translocation to the muscle cell membrane, rather than an increase in the overall GLUT4 protein content in muscle tissue. In the studies conducted by Newman et al. and Van Loon et al. [95,96], exercise was not included, which may explain the lack of observed effects of creatine.

The study by Alves et al. demonstrated that creatine may support the activity of AMPK, showing a positive relationship between changes in AMPK alpha levels and GLUT4 translocation. This may suggest that creatine supplementation could play a role in AMPK alpha signaling, potentially enhancing glucose transport into muscle cells and improving glycemic control in individuals with T2D [93]. Complementary evidence from exercise physiology indicates that physical activity, particularly at moderate to high intensity, also activates AMPK signaling in skeletal muscle [97,98]. Taken together, these findings suggest that both creatine supplementation and exercise may converge on the AMPK–GLUT4 pathway, potentially exerting additive benefits for glucose regulation in T2D.

In summary, creatine supplementation combined with physical exercise appears to enhance glucose uptake and glycemic control more effectively than either intervention alone, largely through GLUT4 translocation and possibly AMPK activation. Clinical trials indicate that this synergy is absent when creatine is used without exercise, underscoring the importance of combining both strategies in T2D management.

## 5. Evidence-Based Safety Profile of Creatine Supplementation

### 5.1. Forms and Dosing Strategies

CrM remains the most extensively studied and widely recommended form of creatine supplementation due to its high purity, stability, and superior bioavailability (~99%) [95,99]. Compared with alternative forms such as creatine ethyl ester or buffered creatine, CrM consistently demonstrates greater muscle uptake and fewer degradation products [99]. Dosing strategies are typically structured around either a rapid “loading” phase or a more gradual “maintenance” approach. In many studies, the initial dosing consists of 20–25 g/day (0.3 g/kg/day), divided into four to five doses over 5–7 days [100,101]. This phase is followed by a maintenance dose of 3–5 g/day (or 0.03 g/kg/day) to sustain elevated intramuscular PCr stores. Notably, several studies have confirmed that daily ingestion of 3–5 g CrM without an initial loading phase can also achieve similar saturation levels over 3–4 weeks, offering a slower but equally effective alternative [101,102,103]. After cessation of supplementation, elevated creatine levels generally return toward baseline over ~4–6 weeks [103].

Emerging evidence supports flexible dosing strategies across demographics, including elderly individuals. For older adults, chronic intake of 0.1 g/kg/day has been shown to enhance muscle function without impairing renal or hepatic markers, underscoring CrM’s favorable safety profile across clinical contexts [104,105,106,107,108,109,110,111]. Moreover, dietary creatine intake (1–3 g/day) remains suboptimal in vegetarians and elderly individuals, further justifying targeted CrM supplementation in these groups [106,107].

### 5.2. Safety Profile and Adverse Effects of Creatine Supplementation in Healthy and Clinical Populations

The safety profile of CrM has been extensively evaluated across age groups and health statuses, with no clinically significant adverse effects reported when used within recommended dosages. According to the International Society of Sports Nutrition (ISSN), creatine is among the most studied and well-tolerated supplements in both exercise and clinical nutrition, supported by over a thousand peer-reviewed publications [84]. Aside from transient weight gain due to increased intracellular water retention, controlled studies have found no consistent or causally substantiated adverse events such as musculoskeletal injury, dehydration, gastrointestinal discomfort, or muscle cramping [84,108].

Despite persistent public concern regarding CrM as a potentially nephrotoxic or hepatotoxic agent, current evidence overwhelmingly indicates that such fears are largely unfounded in both healthy and clinical populations [84,110,112,113]. Early case reports associating CrM with elevated serum creatinine or impaired kidney function often lacked control for confounding variables such as co-ingested supplements or pre-existing renal conditions [114,115]. A seminal long-term observational study by Poortmans et al. followed elite athletes, who self-administered oral creatine (reported in some cases up to 80 g/day) over 10 months to 5 years. No statistically significant differences in plasma creatinine, urea, or albumin levels were observed between creatine users and matched controls, nor were alterations in glomerular filtration rate, tubular reabsorption, or membrane permeability detected; however, randomized trials are needed to confirm long-term safety at high intake levels [116].

These results were corroborated by randomized controlled trials involving patients with neurodegenerative disorders, which consistently reported no CrM-induced renal or hepatic dysfunction [117]. Notably, the transient elevation in serum creatinine sometimes observed following supplementation is now understood as a benign consequence of creatine’s spontaneous conversion to creatinine, rather than impaired filtration [118]. Supporting this, a Mendelian randomization analysis of 1229 participants found no association between systemic creatine levels and six key markers of renal function, reinforcing the notion that excess creatine is efficiently cleared without causing glomerular damage [119]. While some dose-dependent increases in serum or urinary creatinine have been noted, particularly in older adults, these values remained within physiological norms and likely reflect elevated muscle turnover rather than organ dysfunction [120,121].

Further support for creatine’s renal safety comes from animal studies. Ferreira et al. and Baracho et al. demonstrated that even supraphysiological doses of creatine (up to 2 g/kg/day) in Wistar rats did not impair glomerular filtration, renal plasma flow, or liver integrity [109,110]. Similarly, clinical trials involving human participants consuming up to 30 g/day of creatine for as long as five years reported no meaningful changes in renal or hepatic markers [111,112,116].

Reinforcing this body of evidence, a comprehensive systematic review and meta-analysis of 685 randomized human trials concluded that CrM supplementation does not increase adverse event incidence compared to placebo. Across over 26,000 participants, adverse effects were reported by 4.60% of creatine users versus 4.21% in the placebo group, a non-significant difference (*p* = 0.828). No elevated rates of gastrointestinal symptoms (*p* = 0.820), muscle cramping (*p* = 0.085), or renal abnormalities were observed. These findings were consistent across children, older adults, clinical patients, and athletes, confirming CrM’s broad tolerability across diverse physiological conditions. Importantly, even high-dose and long-duration protocols did not alter safety outcomes, affirming CrM’s role in evidence-based nutritional strategies [122].

### 5.3. Safety and Metabolic Tolerability of Creatine Monohydrate in Older Adults with T2D and Sarcopenia

Emerging evidence suggests that CrM supplementation is safe for use in older individuals with T2D, a group often affected by sarcopenia and multimorbidity. Gualano et al. demonstrated that 12 weeks of creatine intake at a dosage of 5 g/day in patients with T2D did not adversely affect renal function, as measured by albuminuria, proteinuria, blood urea nitrogen, and estimated creatinine clearance, indicating preserved kidney integrity under supplementation [123].

In a cohort of frail older adults (n = 9; 70 ± 5 years), Collins et al. reported that combining 5 g/day of CrM with protein intake during 14 weeks of resistance training did not affect serum levels of renal or hepatic markers, including blood urea nitrogen, creatinine, bilirubin, alkaline phosphatase, gamma-glutamyltransferase, alanine aminotransferase, aspartate aminotransferase, or creatine kinase, suggesting no detectable organ toxicity in this population [94].

Similarly, long-term studies in postmenopausal women receiving creatine at either 1 g/day or 0.1 g/kg/day for 12 months revealed no detrimental changes in markers of liver or kidney function, including urinary microalbumin, urine protein, and glomerular filtration indicators [124]. Additionally, no signs of cytotoxicity were observed, as evidenced by unchanged urinary formaldehyde levels in elderly men undergoing whole-body resistance training for 10 weeks while receiving CrM (0.1 g/kg/day) [105].

Building on emerging evidence of creatine’s favorable safety profile in clinical populations, a systematic review by de Sousa et al. critically assessed the efficacy and tolerability of CrM in individuals with T2D. The review synthesized findings from three randomized controlled trials involving 87 participants, in which CrM was compared to placebo, metformin, and glibenclamide. Across studies, CrM supplementation led to modest yet favorable reductions in fasting blood glucose and HbA1c, without exceeding the glycemic efficacy of conventional pharmacotherapies. Importantly, none of the trials reported any serious adverse events attributable to CrM, reinforcing its metabolic safety in this high-risk population. However, due to small sample sizes and heterogeneity in study design, the authors urged caution in interpretation and highlighted the need for large-scale, long-term trials [125].

### 5.4. Creatine Co-Supplementation: Pharmacokinetic Interactions and Clinical Implications

The safety and efficacy of CrM are well-established; however, its concurrent administration with other dietary components or pharmaceutical agents introduces pharmacokinetic and pharmacodynamic complexities that merit critical evaluation in both athletic and clinical settings. Caffeine remains one of the most frequently co-consumed compounds with creatine, and its interaction profile is paradoxical. A systematic review by Elosegui et al. demonstrated that acute co-administration of caffeine and creatine does not impair caffeine’s ergogenic effects, yet chronic simultaneous supplementation may attenuate creatine’s performance-enhancing properties, possibly via antagonistic effects on muscle relaxation dynamics and calcium handling [126]. Trexler et al. further support this hypothesis, indicating that ergogenic blunting occurs without changes in creatine’s plasma kinetics, suggesting a non-systemic mechanism [127]. Vanakoski et al. also found no significant changes in maximum plasma concentration (Cmax), time to reach maximum plasma concentration (Tmax), or half-life of creatine when co-ingested with caffeine, reinforcing the likelihood of local (muscle-level) rather than systemic interactions [128].

Carbohydrate co-ingestion, on the other hand, has been shown to significantly enhance creatine uptake via insulin-mediated pathways. Green et al. and Steenge et al. demonstrated that combining CrM with simple sugars reduced plasma creatine area under the curve (AUC) and urinary excretion, while increasing intramuscular retention—effects attributed to insulin’s role in upregulating muscle creatine transporter activity. These observations confirm that co-ingestion with carbohydrates enhances bioavailability and muscular deposition of creatine through endocrine mechanisms. This has clinical relevance in populations with altered insulin sensitivity, such as individuals with metabolic syndrome or T2D [129,130].

The form of creatine ingested may also influence efficacy; for example, creatine ethyl ester—despite claims of improved bioavailability via reduced hydrophilicity and transporter-independent uptake—undergoes rapid nonenzymatic conversion to creatinine and does not meaningfully increase intramuscular PCr stores [102].

Beyond nutrient–nutrient interactions, emerging data point to possible drug–creatine interactions that could have important clinical implications. According to the drug–drug interaction database (DDInter), CrM has the potential to interact with several pharmaceutical agents, including pemetrexed, entecavir, cimetidine, trimethoprim, and probenecid. Co-administration of CrM with these compounds, particularly in older adults, may elevate both serum drug concentrations and serum creatinine levels, raising important concerns about altered renal clearance and potential misinterpretation of kidney function biomarkers in polypharmacy contexts [107]. These signals require clinical verification; monitor renal function and consider drug pharmacokinetics in polypharmacy, especially in older adults.

In summary, CrM remains a safe and effective supplement when used in isolation, but co-administration with other agents, such as caffeine or specific pharmaceutical drugs, may modulate its efficacy or confound biomarker interpretation. Future studies should aim to standardize co-supplementation protocols and evaluate population-specific pharmacodynamic responses, especially in older adults and those with comorbidities or complex medication regimens.

## 6. Conclusions

Current evidence from mechanistic studies and clinical trials indicates that creatine supplementation, principally as creatine monohydrate, combined with structured resistance and/or aerobic exercise, is a promising adjunct for the prevention and management of T2D. The combined intervention consistently improves muscle glucose handling via enhanced GLUT4 translocation, greater muscle glycogen storage, and possible AMPK modulation and augments muscle mass and strength. These effects plausibly translate into improved glycemic control and mitigation of sarcopenia-related metabolic decline. Importantly, benefits reported to date are most robust when creatine is administered together with exercise; creatine alone yields smaller and less consistent glycemic effects.

The safety profile of creatine at commonly recommended regimens (for example, 3–5 g/day or standard short loading protocols) appears favorable, including in older adults and persons with T2D. Randomized and observational data do not support clinically meaningful nephrotoxicity or hepatotoxicity in otherwise stable populations. Long-term evidence for very high chronic dosing (e.g., sustained > 20–30 g/day) is limited, and such regimens should be approached cautiously. A clinically relevant issue is the dual interpretation of creatinine: it is both the metabolic breakdown product influenced by supplementation and a marker of muscle mass. Modest rises in serum creatinine after creatine intake commonly reflect increased creatine–creatinine conversion or greater muscle mass/turnover rather than true renal injury. Conversely, low creatinine may signal reduced muscle mass and sarcopenia. To avoid misclassification, combine biochemical monitoring with direct measures of muscle mass and function (e.g., DXA or bioimpedance, handgrip strength, physical performance tests) and consider creatinine-independent renal markers (e.g., cystatin C) when clinically appropriate. Pharmacokinetic and pharmacodynamic interactions (signals reported with certain drugs, caffeine, or co-ingested nutrients) warrant clinician awareness but require further clinical verification. In patients with polypharmacy or pre-existing kidney disease, perform baseline renal assessment, monitor function periodically, and interpret creatinine-based GFR estimates with caution.

Research priorities remain: adequately powered, long-term randomized controlled trials in people with T2D, particularly older adults and those with sarcopenia, are needed to define optimal dosing and duration, confirm durability of metabolic and functional benefits, and establish safety in multimorbid and polypharmacy contexts. Trials should include mechanistic endpoints (GLUT4, AMPK, muscle glycogen, satellite cell activity), standardized co-supplementation protocols, and creatinine-independent renal assessment where feasible.

Overall, the reviewed evidence supports the considered use of creatine supplementation combined with exercise as a safe and potentially effective adjunct to standard T2D prevention and management strategies, provided that interventions are individualized and appropriate monitoring is implemented.

## Figures and Tables

**Table 1:** Summary of randomized and controlled trials of creatine + exercise, including studies in T2D, older adults, and healthy volunteers.

Study	Design	N	Population (Mean Age)	Intervention—Creatine (Form, Dose, Scheme) + Exercise (Type, Freq, Intensity)	Comparator	Duration	Metabolic Outcomes (Direction; Significance)	Muscle Outcomes (Lean Mass, Strength, Function)	Safety/Renal Markers
Op’t Eijnde B et al., 2001 [23]	DBPC RCT	22	Healthy volunteers (immobilization model)	CrM during immobilization + rehabilitation; resistance rehabilitation	Placebo	2 weeks immobilization + 10 weeks rehabilitation	↑ GLUT4 preservation/↑ GLUT4 during rehabilitation(vs placebo)	↑ GLUT4 and glycogen recovery with retraining	No safety signals reported
Derave W et al., 2003 [90]	DBPC RCT	33	Healthy adults (one-leg immobilization)	CrM ± protein during immobilization/retraining; localized resistance retraining	Placebo/creatine arms	2 weeks immobilization + 6 weeks retraining	↑ glycogen and improved GLUT4 recovery when CrM + protein + retraining	↑ muscle glycogen; ↑ GLUT4 with activity	No adverse renal effects reported
Gualano B et al., 2008 [91]	RCT DBPC (aerobic trial)	22	Sedentary healthy males	CrM + moderate-intensity aerobic training	Placebo	12 weeks	Improved OGTT glucose response (significant); no change fasting insulin/HOMA-IR	↑ glycogen (no GLUT4 protein change)	No safety signals reported
Gualano B et al., 2011 [22]	RCT DBPC (T2D)	25	Adults with T2D	CrM 5 g/day + combined aerobic + resistance (3×/wk)	Placebo	12 weeks	↓ HbA1c and ↓ meal glycemia (significant); insulin/C-peptide unchanged	Functional/muscle benefits reported with training	No detrimental changes in albuminuria/BUN/creatinine
Oliveira CLP et al., 2020 [92]	RCT DBPC pilot	NR (small)	Community-dwelling older adults	CrM + resistance training (dose per paper)	Placebo + resistance training	12 weeks	No significant change in fasting insulin or HOMA-IR	Variable muscle improvements; inconsistent additive benefit	No renal/hepatic adverse effects reported
Alves CR et al., 2012 [93]	Mechanistic RCT/trial	NR	Individuals with T2D/mechanistic samples	CrM supplementation (dose per paper) ± exercise	Placebo/control	Short term	AMPKα activation signals correlated with GLUT4 translocation (suggested)	Not primary for clinical muscle endpoints	No safety concerns reported
Collins J et al., 2016 [94]	Pilot RCT/cohort	9	Frail older adults (70 ± 5 y)	CrM 5 g/day + protein + resistance training	Protein + resistance training	14 weeks	Metabolic markers not significantly altered	↑ Strength; no adverse muscle damage markers	No changes in renal/hepatic markers reported
Newman JE et al., 2003 [95]	RCTs (negative/mixed)	Small samples	Healthy males	CrM (standard protocols) ± training	Placebo	Short term	No consistent effects on insulin sensitivity/fasting insulin/HOMA-IR	↑ glycogen (van Loon) but no GLUT4 protein increase; mixed strength effects	No safety signals
van Loon LJ et al., 2004 [96]	NRCT DBPC	Small samples	Healthy males	CrM (standard protocols) ± training	Placebo	6 weeks	No consistent effects on fasting insulin	↑ glycogen but no GLUT4 protein increase	No safety signals

Footnotes: DBPC, double-blind placebo-controlled; RCT, randomized controlled trial; CrM, creatine monohydrate; GLUT4, glucose transporter type 4; OGTT, oral glucose tolerance test; HOMA-IR, homeostatic model assessment of insulin resistance; T2D, type 2 diabetes; AMPKα, AMP-activated protein kinase alpha subunit; BUN, blood urea nitrogen; NR, not reported; y, years; g/day, grams per day; ×/wk, times per week. Arrows (↑, ↓) indicate increase or decrease, respectively. Only two trials were conducted in patients with T2D; the remaining studies involved older adults, healthy volunteers, or experimental immobilization models.

## Abbreviations

The following abbreviations are used in this manuscript:

T2D	Type 2 Diabetes
IDF	International Diabetes Federation
HbA1c	Glycated Hemoglobin
PCr	Phosphocreatine
CK	Creatine Kinase
CRTR	Creatine Transporter
ROS	Reactive Oxygen Species
AMPK	AMP-Activated Protein Kinase
PGC-1α	Peroxisome Proliferator-Activated Receptor Gamma Coactivator 1-Alpha
NF-κB	Nuclear Factor Kappa B
p38 MAPK	P38 Mitogen-Activated Protein Kinase
TNF-α	Tumor Necrosis Factor Alpha
IL-1β	Interleukin-1 Beta
STAT1/3	Signal Transducer And Activator Of Transcription 1/3
IGF-1	Insulin-Like Growth Factor 1
GH	Growth Hormone
mTORC1	Mechanistic Target Of Rapamycin Complex 1
GLUT4	Glucose Transporter Type 4
Akt/PKB	Protein Kinase B
Rac1	Ras-Related C3 Botulinum Toxin Substrate 1
CaMK	Calcium/Calmodulin-Dependent Protein Kinase
IRS	Insulin Receptor Substrate
WHO	World Health Organization
OGTT	Oral Glucose Tolerance Test
HOMA-IR	Homeostatic Model Assessment Of Insulin Resistance
CrM	Creatine Monohydrate
ISSN	International Society Of Sports Nutrition
Cmax	Maximum Plasma Concentration
Tmax	Time To Reach Maximum Plasma Concentration
AUC	Area Under The Curve
CEE	Creatine Ethyl Ester
DDInter	Drug–Drug Interaction Database
